# The Association Between Schizophrenia Risk Variants and Creativity in Healthy Han Chinese Subjects

**DOI:** 10.3389/fpsyg.2019.02218

**Published:** 2019-10-01

**Authors:** Dan Wang, Tingting Guo, Qi Guo, Shun Zhang, Jinghuan Zhang, Jing Luo, GeseDNA Research Team, Leilei Zhang

**Affiliations:** Beijing Gese Technology Co., Ltd., Beijing, China; ^1^Beijing Key Laboratory of Learning and Cognition, Department of Psychology, The Collaborative Innovation Center for Capital Education Development, Capital Normal University, Beijing, China; ^2^Beijing Gese Technology Co., Ltd., Beijing, China; ^3^Department of Psychology, Shandong Normal University, Jinan, China

**Keywords:** schizophrenia, creativity, genes, divergent thinking, risk variants

## Abstract

Although previous evidence has suggested that there is a genetic link between schizophrenia and creativity, the specific genetic variants that underlie the link are still largely unknown. To further explore the potential genetic link between schizophrenia and creativity, in a sample of 580 healthy Han Chinese subjects, this study aimed to (1) validate the role of Neuregulin 1 (NRG1) rs6994992 (one schizophrenia risk variant that has been previously linked to creativity in the European population) in the relationship between schizophrenia and creativity and (2) explore the associations between 10 other schizophrenia risk variants and creativity. For NRG1 rs6994992, the result validated its association with creativity measures. However, since NRG1 rs6994992 is not a schizophrenia risk variant in the Han Chinese population, the validated association suggested that ethnic difference may exist in the relationship between NRG1 rs6994992, schizophrenia and creativity. For other schizophrenia risk variants, the result only demonstrated a nominal association between ZNF536 rs2053079 and creativity measures which would not survive correction for multiple testing. No association between polygenic risk score for these 10 schizophrenia risk variants and creativity measures was observed. In conclusion, this study provides limited evidence for the associations between these schizophrenia risk variants and creativity in healthy Han Chinese subjects. Future studies are warranted to better understand the potential genetic link between schizophrenia and creativity.

## Introduction

John Forbes Nash, the Father of Game Theory, had suffered from schizophrenia. He once said, “I wouldn’t have had good scientific ideas if I had thought more normally”. Along similar lines, many of the creative luminaries throughout history (e.g., Pink Floyd frontman Syd Barrett) have been associated with schizophrenia (Neel Burton, 2015). Do these anecdotes suggest that, at least in some cases, there is a link between schizophrenia and creativity?

 Actually, the relationship between schizophrenia and creativity has been of particular interest for psychologists for a long time, and previous studies has provided mixed evidence reporting both positive and negative relationships. While some studies shown that people with schizophrenia are more creative and creative people are often associated with an elevated level of schizotypy and psychosis proneness (Burch et al., 2006; Karksson, 2010; Fink et al., 2014), there has been evidence showing the opposite results (Nemoto et al., 2005; Kyaga et al., 2013; Son et al., 2015). To further clarify the relationship, Acar et al. (2018) recently conducted a meta-analysis with 200 effect sizes obtained from 42 studies, and the result demonstrated that schizophrenia and creativity seem to have an inverted-U relationship, in which mild expression of schizophrenia symptoms (one or two minor signs of symptoms) may facilitate creativity but more severe signs of the symptoms would be detrimental to creativity.

Although the link between schizophrenia and creativity has received empirical support, there has been one important question remains to be answered; that is, how and by what mechanisms schizophrenia and creativity are correlated. The “shared vulnerability model” may help to answer this question. Based on findings from neuroimaging and genetic studies, Carson (2011) proposed the “shared vulnerability model” to explain the relationship between psychopathology and creativity. According to the model, psychiatric disorders (e.g., schizophrenia) and creativity may share specific overlapping cognitive processes; and more importantly, there might be a genetic link between psychiatric disorders and creativity. This model provided a new perspective to understand the link between schizophrenia and creativity, and highlighted the potential effects of schizophrenia risk variants on creativity.

Previous genetic epidemiological studies have indicated that schizophrenia is highly heritable. Due to the great attention that schizophrenia has attracted and the availability of large samples, a series of risk variants have recently been identified (Shi et al., 2009, 2011; Stefansson et al., 2009; Lee et al., 2013; Li et al., 2017). According to the “common disease/common variant” hypothesis, the risk alleles of these risk variants are also carried by healthy subjects in general populations and may thus impact brain development and cognitive functions of normal healthy subjects.

Consistent with this hypothesis, by examining the association between Neuregulin 1 gene (*NRG1*) and creativity, Kéri (2010) investigated for the first time the association between schizophrenia risk variants and creativity in healthy subjects. In a sample of healthy European subjects, Kéri (2010) showed that the TT genotype of *NRG1* rs6994992 polymorphism, which has been previously related to schizophrenia risk (Wei et al., 2007; Kéri et al., 2009), was associated with higher creative achievements. This study provided the first direct evidence for the association between schizophrenia risk variant and creativity in healthy subjects, and suggested that *NRG1* rs6994992 might be among the many risk variants that underlie the genetic link between schizophrenia and creativity. However, it is worth noting that, although *NRG1* rs6994992 has been widely recognized as an important schizophrenia risk variant (Li et al., 2006; Jeremy et al., 2008; Buonanno, 2010), recent studies have also suggested an ethnic difference in the effect of *NRG1* rs6994992 on schizophrenia (i.e., the relationship between *NRG1* rs6994992 and schizophrenia was mainly observed in the European population, but not in the Han Chinese population) (Zhao et al., 2004; Mostaid et al., 2016). Considering this fact, to further validate whether *NRG1* rs6994992 underlies the genetic link between schizophrenia and creativity, it is necessary to reevaluate the association between *NRG1* rs6994992 and creativity in other populations. Thus, the first aim of this study was to clarify the relationship between *NRG1* rs6994992, schizophrenia and creativity by validating the association between *NRG1* rs6994992 and creativity in healthy subjects of Han Chinese population in which *NRG1* rs6994992 is not a schizophrenia risk variant.

Moreover, as mentioned above, although attempts have been made to directly investigate the association between schizophrenia risk variants and creativity, this subject of research has not received enough attention in the literature. Until now, besides *NRG1* rs6994992, the associations between other schizophrenia risk variants and creativity have not yet been explored, and it is therefore still unknown whether there have been other schizophrenia risk variants that underlie the genetic link between schizophrenia and creativity. With this in mind, the second aim of this study was to explore whether other reported schizophrenia risk variants are associated with creativity in healthy Han Chinese subjects.

## Materials and Methods

### Subjects

Subjects aged 18–55 years predominantly from Beijing, China, were recruited through Internet and social media advertisements. The initial inclusion criteria were: (1) written informed consent; (2) parents and grandparents were Han Chinese; (3) aged 18–55 years; (4) no self-reported history of neurological or psychiatric disorders (including substance abuse or dependence); (5) no current psychoactive medications. Recruitment began in November 2017 and ended in September 2018. A total of 624 subjects were enrolled into this study. Among these eligible subjects, 44 were excluded because of discordant gender information, low quality genotyping data (genotyping failure in more than 1% of genetic variants in Illumina Infinium Global Screening Array), genetic relatedness with other subjects, or missing phenotype data, leaving 580 subjects (310 males, 270 females) as the final sample. The subjects had a mean age of 26.43 years (*SD* = 5.82). Written informed consent forms were obtained from all subjects. This study was approved by the Ethics Committee of Psychology Department of Capital Normal University.

### Measures of Creativity

Two alternative-uses tasks (“comb” and “toothpick”) were used in the present study to measure creativity. In these tasks, subjects were given 3 min to list as many uses as possible for each common object. Each participant’s responses were scored for the three core dimensions of creativity (fluency, flexibility, and originality). Specifically, the fluency score is the number of responses. The flexibility score is the number of categories used in one’s responses. The originality score is the number of unusual responses (given by less than 5% of all subjects). Finally, a total score was calculated by summing the scores of all three dimensions. Three trained graduate students from Capital Normal University rated all the responses.

### Genetic Variants Selection and Genotyping

Besides *NRG1* rs6994992, 10 other schizophrenia risk variants were also selected and genotyped in this study. The selection of these schizophrenia risk variants was mainly based on previous association studies of schizophrenia. The inclusion criteria was as follows: (a) risk variants that have been implicated in recent GWAS or meta-analysis of schizophrenia with the Asian population or the Han Chinese population (Yuan et al., 2012; Li et al., 2017; Liu et al., 2017; Xiao et al., 2017; Yang et al., 2018); (b) minor allele frequency (MAF) ≥5% in the Han Chinese population based on the 1000 Genomes Project database; (c) known or promising importance in neural developments and brain functions related to creativity.

Genomic DNA extracted from saliva samples was genotyped using an Illumina Infinium Global Screening Array (Illumina, San Diego, CA, United States) at Beijing Gese Technology Co., Ltd., All 11 genetic variants passed the quality control criteria of call rate >95%, MAF > 10%, and consistency with Hardy-Weinberg equilibrium (*p* > 0.05, Fisher exact test). The overview of information on all studied schizophrenia risk variants is provided in Table 1.

**TABLE 1:** Characteristics of the genotyped schizophrenia risk variants.

**Chr**	**Position^a^**	**Variant**	**Gene**	**Allele (minor/major)**	**Risk allele^*b*^**	**Odds ratio^*c*^**	**References**	**MAF (%)**
1	30432219	rs4949526	—	T/C	T	1.044	Li et al., 2017	25
1	167903079	rs10489202	MPC2	T/G	T	1.161	Yang et al., 2018	16
1	177280121	rs6670165	—	T/C	T	1.068	Li et al., 2017	14
2	233743109	rs778371	C2orf82	G/A	G	1.074	Li et al., 2017	12
6	108983527	rs9398171	FOXO3	C/T	C	1.082	Li et al., 2017	26
8	4180844	rs10503253	CSMD1	A/C	A	1.062	Liu et al., 2017	30
8	31495581	rs6994992	NRG1	C/T	—	—	Keri et al., 2009	40
10	62179812	rs10994336	ANK3	T/C	T	1.19	Yuan et al., 2012	22
10	104775908	rs7914558	CNNM2	G/A	G	1.059	Xiao et al., 2017	39
16	9946319	rs9922678	GRIN2A	A/G	A	1.041	Li et al., 2017	13
19	30987423	rs2053079	ZNF536	G/A	G	1.032	Li et al., 2017	18

*^*a*^Physical position based on NCBI Genome Build GRCh37/hg19. ^*b*^Risk allele in the Han Chinese population. Since *NRG1* rs6994992 is not a schizophrenia risk variant in the Han Chinese population, no risk allele was present. ^*c*^Odds ratio obtained from recent GWAS or meta-analysis of schizophrenia with the Asian population or the Han Chinese population. For rs778371, rs9398171, and rs2053079, the original allele reported in the literature was protective allele with odds ratio of less than 1. To calculate polygenic risk score, the odds ratio was inverted and the alternate allele was used as the risk allele.*

### Statistical Analysis

To adjust for the possible presence of population stratification, principal component analysis (based on quality controlled and linkage disequilibrium pruned data of 132,888 variants from Illumina Infinium Global Screening Array) was applied using EIGENSOFT v7.2.1 software (Price et al., 2006). The first three principal components as well as gender and age (Matud et al., 2007; Palmiero et al., 2014, 2017; Palmiero, 2015) were included as covariates in all the genetic analyses mentioned below. Single variant analysis under additive genetic model was performed using linear regression in PLINK v1.07 software (Purcell et al., 2007). Polygenic risk score for schizophrenia risk variants was calculated by adding a given value for each variant with an associated risk allele. Weighted risk score was calculated using the scoring option implemented in PLINK. Each score was given a weight according to the natural log transformed odds ratio provided for each risk variant. Odds ratios for risk variants were obtained from recent GWAS or meta-analysis of schizophrenia with the Asian population or the Han Chinese population (see reference in Table 2). The unweighted risk score was calculated as the number of risk alleles counted for each subject. *NRG1* rs6994992 was excluded from this analysis because it is not a risk variant in the Han Chinese population. Linear regression was used to test the associations between polygenic risk score and creativity measures. Power calculation for single variant analysis was performed using the QUANTO program (Knight, 2004).

**TABLE 2:** Descriptive statistics and inter-correlations.

**Measure**	**M ± SD**			**Fluency**	**Flexibility**	**Originality**	**Total**	**Age**
						
	**Male**	**Female**	**Total**					
Fluency	5.42 (3.31)	5.59 (3.61)	5.50 (3.45)	—	0.958^∗∗^	0.511^∗∗^	0.980^∗∗^	0.072
Flexibility	3.77 (2.40)	3.91 (2.52)	3.83 (2.45)		—	0.652^∗∗^	0.991^∗∗^	0.073
Originality	4.69 (0.83)	4.73 (0.75)	4.71 (0.79)			—	0.656^∗∗^	0.013
Total	13.89 (6.12)	14.23 (6.58)	14.00 (6.33)				—	0.069
Age	26.34 (5.85)	26.54 (5.79)	26.43 (5.82)					—

*^∗∗^*p* < 0.01.*

## Results

Descriptive statistics and inter-correlations are shown in Table 2. Significant correlations were observed between the four indexes of creativity measures, while no significant effect of gender and age was observed.

Table 3 summarizes the results of the single variant analysis and polygenic risk score analysis. Two SNPs showed nominal associations with creativity measures. Specifically, the *NRG1* rs6994992 showed nominal associations with fluency (effect size *R*^2^ = 0.009), flexibility (effect size *R*^2^ = 0.007) and the total score (effect size *R*^2^ = 0.008), with the T allele being associated with higher scores; the *ZNF536* rs2053079 showed a nominal association with originality (effect size *R*^2^ = 0.007), with the A allele being associated with higher scores. Power calculation showed that, for common genetic variants (MAF > 10%) under the additive model, our sample has 80% power to detect effect size (*R*^2^) greater than 0.0135 (with α = 0.05). As shown in Table 3, the result of polygenic risk score analysis showed that neither the weighted polygenic risk score nor the unweighted polygenic risk score was associated with the four indexes of creativity measures.

**TABLE 3:** Results of single variant analysis and polygenic risk score analysis.

	**Fluency**			**Flexibility**			**Originality**			**Total**		
				
	**β (*SE*)**	***t***	***p***	**β (*SE*)**	***t***	***p***	**β (*SE*)**	***t***	***p***	**β (*SE*)**	***t***	***p***
rs4949526	0.007 (0.042)	0.173	0.863	−0.001(0.042)	–0.030	0.976	−0.03(0.042)	–0.718	0.473	0.000 (0.042)	–0.007	0.994
rs10489202	0.032 (0.042)	0.760	0.448	0.035 (0.042)	0.834	0.405	0.037 (0.042)	0.893	0.372	0.035 (0.042)	0.849	0.396
rs6670165	0.023 (0.042)	0.556	0.578	0.027 (0.042)	0.646	0.518	−0.012(0.042)	–0.280	0.780	0.022 (0.042)	0.518	0.605
rs778371	−0.02(0.042)	–0.470	0.639	−0.018(0.042)	–0.437	0.662	0.006 (0.042)	0.144	0.886	−0.017(0.042)	–0.407	0.684
rs9398171	0.026 (0.042)	0.629	0.530	0.026 (0.042)	0.634	0.526	0.008 (0.042)	0.201	0.840	0.026 (0.042)	0.613	0.540
rs10503253	0.023 (0.042)	0.555	0.579	0.034 (0.042)	0.806	0.421	0.039 (0.042)	0.928	0.354	0.030 (0.042)	0.731	0.465
**rs6994992**	**−0.094 (0.041)**	**−2.257**	**0.024**	**−0.083 (0.042)**	**−1.996**	**0.046**	−0.06(0.042)	–1.429	0.154	**−0.091 (0.042)**	**−2.182**	**0.030**
rs10994336	−0.001(0.042)	–0.014	0.989	0.007 (0.042)	0.174	0.862	0.041 (0.042)	0.971	0.332	0.008 (0.042)	0.182	0.856
rs7914558	−0.017(0.042)	–0.405	0.686	−0.015(0.042)	–0.356	0.722	0.044 (0.042)	1.050	0.294	−0.009(0.042)	–0.227	0.821
rs9922678	0.017 (0.041)	0.414	0.679	0.008 (0.041)	0.202	0.840	−0.036(0.042)	–0.858	0.391	0.008 (0.041)	0.196	0.845
**rs2053079**	0.036 (0.041)	0.871	0.384	0.057 (0.041)	1.375	0.170	**0.083 (0.041)**	**2.006**	**0.045**	0.052 (0.041)	1.259	0.209
Polygenic risk score(weighted)	0.031 (0.042)	0.745	0.456	0.041 (0.042)	0.997	0.319	0.065 (0.042)	1.558	0.120	0.041 (0.042)	0.988	0.324
Polygenic risk score (unweighted)	0.039 (0.041)	0.930	0.353	0.049 (0.042)	1.187	0.236	0.062 (0.042)	1.487	0.138	0.048 (0.041)	1.153	0.249

**NRG1* rs6994992 was not included in the polygenic risk score analysis, since it is not a schizophrenia risk variant in the Han Chinese population. The nominally significant associations are indicated in bold.*

## Discussion

Although the debate over the relationship between schizophrenia and creativity has continued in the literature, recent evidence has suggested that there might be a genetic link between schizophrenia and creativity. In this study, to explore the potential genetic link, 11 schizophrenia risk variants were genotyped and tested for their associations with creativity in a sample of healthy Han Chinese subjects.

Of the 11 schizophrenia risk variants, only *NRG1* rs6994992 has been previously examined for its association with creativity (Kéri, 2010). The *NRG1* encodes a key developmental growth factor that involved in neural development, neurotransmission and synaptic plasticity (Harrison and Law, 2006). The rs6994992 polymorphism is located in the type IV promoter region of *NRG1* and the T allele of rs6994992 has been shown to be associated with increased transcript levels of type IV NRG1 in brains of both healthy and schizophrenic patients (Law et al., 2006). To demonstrate the role of rs6994992 in schizophrenia, many studies have been conducted to investigate the association between NRG1 rs6994992 and schizophrenia in different ethnic populations (e.g., Stefansson et al., 2002, 2003; Zhao et al., 2004; Petryshen et al., 2005; Li et al., 2006). Although the overall results generally supported the association between NRG1 rs6994992 and schizophrenia, there has also been evidence suggested an ethnic difference in the relationship between NRG1 rs6994992 and schizophrenia, with the association mainly being present in the European populations (Li et al., 2006; Mostaid et al., 2016; Jagannath et al., 2017). Specially, in the Asian population, several studies have suggested that NRG1 rs6994992 may not be a schizophrenia risk variant. For example, using both case-control and family-based designs, Zhao et al. (2004) and Li et al. (2006) showed that NRG1 rs6994992 was not associated with schizophrenia in the Han Chinese population. In samples of the Japanese and the Korean populations, Iwata et al. (2003) and Kim et al. (2006) showed that NRG1 rs6994992 was not associated with schizophrenia. Similar results were also obtained by three meta-analysis studies examining the association between NRG1 and schizophrenia (Li et al., 2006; Mostaid et al., 2016; Jagannath et al., 2017). Moreover, since schizophrenia and other psychiatric disorders may have shared genetic etiology (Berrettini, 2000; Bramon and Sham, 2001; Cardno et al., 2002; Craddock et al., 2009; Huang et al., 2010; Cross-Disorder Group of the Psychiatric Genomics Consortium Lee et al., 2013), several studies have also investigated the association between NRG1 rs6994992 and other psychiatric disorders such as bipolar disorder (e.g., Green et al., 2005; Cassidy et al., 2006; Thomson et al., 2007; Georgieva et al., 2008; Goes et al., 2009). While findings from these studies tended to support for the role of NRG1 rs6994992 (mainly as part of haplotype) in bipolar disorder, all these studies were conducted in the European population. According to the best of our knowledge, only a few studies have investigated the association between NRG1 and bipolar disorder in the Asian population (including the Han Chinese population), and none examined NRG1 rs6994992 (e.g., Crisafulli et al., 2013; Cao et al., 2014; Wen et al., 2016). Thus, whether NRG1 rs6994992 was also related to bipolar disorder in the Asian population remains to be elusive.

As for the relationship between *NRG1* rs6994992 and creativity, Kéri (2010) first found that *NRG1* rs6994992 was associated with creativity in a sample of healthy European subjects, and suggested that *NRG1* rs6994992 may be one risk variant that underlies the genetic link between schizophrenia and creativity. In this study, the association between *NRG1* rs6994992 and creativity was replicated in a sample of healthy Han Chinese subjects. However, since *NRG1* rs6994992 was not a schizophrenia risk variant in the Han Chinese population, it was expected that no association between *NRG1* rs6994992 and creativity would observed in our sample if *NRG1* rs6994992 underlies the genetic link between schizophrenia and creativity. Therefore, the results of this study raised new questions about the role of *NRG1* rs6994992 in the relationship between schizophrenia and creativity, and suggested ethnic differences may also exist in the relationship between *NRG1* rs6994992, schizophrenia and creativity.

Ethnic difference has particular relevance for understanding the genetic basis of complex diseases and traits since many genes that are known to have been affected by natural selection are functionally important (Pickrell et al., 2009; Hofer et al., 2012; Field et al., 2016). The level of ethnic difference shown by diverse ethnic populations is related to allele frequency differences of genetic variants (Lan et al., 2007; Mattei et al., 2009). Because different populations are subject to distinct environments, certain environmental conditions may act through selective pressure to alter the frequencies of genetic variants and result in population-specific allele frequencies (Cavalli-Sforza et al., 1994; Chikhi et al., 1998; Cavalli-Sforza and Feldman, 2003; Myles et al., 2008; Hancock et al., 2011; Corona et al., 2013). Several studies have highlighted the importance of population-specific allele frequencies in gene expression and suggested that allele frequency differences of regulatory genetic variants (e.g., *NRG1* rs6994992 located in promoter region of *NRG1*) may significantly influence gene expression and thus lead to differences in the genetic basis of complex diseases and traits across ethnic populations (Goddard et al., 2000; Ioannidis et al., 2004; Spielman et al., 2007; Myles et al., 2008).

In the case of *NRG1* rs6994992, given that significant allele frequency differences were observed for the risk T allele between the Han Chinese population (60%) and the European population (37%), it is possible that such population-specific allele frequencies may account for some of the reasons for the ethnic difference in the relationship between *NRG1* rs6994992, schizophrenia and creativity (i.e., allele frequency may moderate the relationship between *NRG1* rs6994992, schizophrenia and creativity). Based on our results and the high frequency of the T allele in the Han Chinese population, it seems that while the association between *NRG1* rs6994992 and schizophrenia is more subject to allele frequency of the T allele (the risk for schizophrenia mediated by the T allele might be masked by the high frequency of the T allele in the Han Chinese population), the association between *NRG1* rs6994992 and creativity is not affected by allele frequency of the T allele and thus more consistent across different ethnic populations. And this consistency is supported by recent neuroimaging findings that *NRG1* rs6994992 was associated with creativity-related brain structures and function in healthy subjects of different ethnic populations. For example, in healthy European subjects it has been shown that *NRG1* rs6994992 was associated with differences in frontal brain structures in both gray and white matter (Hall et al., 2006; Mcintosh et al., 2008; Knickmeyer et al., 2014). And more recently, in healthy Chinese subjects *NRG1* rs6994992 has been shown to be related to both structures and functional connectivity of frontal and temporal lobes, hippocampus and angular gyrus (Zhang et al., 2019).

Besides *NRG1* rs6994992, 10 other schizophrenia risk variants were also investigated in this study and the results showed that only *ZNF536* rs2053079 was nominally associated with creativity. Because *ZNF536* rs2053079 has not been previously examined for its association with creativity, our result provides the first evidence for the association of this schizophrenia risk variant with creativity in healthy subjects. *ZNF536* encodes a highly conserved novel zinc finger protein that is most abundant in the brain and negatively regulates neuron differentiation (Qin et al., 2009). Recent findings have suggested that *ZNF536* may play an essential role in development of forebrain neurons (Thyme et al., 2019). The rs2053079 polymorphism is located in the intron region of *ZNF536*. Although the exact biological function of rs2053079 has not been established, this intron variant has been repeatedly implicated in recent large-scale GWAS of schizophrenia and creativity-related cognitive functions (Davies et al., 2018; Lee et al., 2018). Based on this evidence, it is possible that *ZNF536* rs2053079 may play a role in the relationship between schizophrenia and creativity. However, it should also be noted that, owing to the very limited statistical power in this study, *ZNF536* rs2053079 only showed nominal association with creativity. Since underpowered studies have increased risk of false positive results, this result may suffer from type I error. This coincides with the fact that the nominal associations between *ZNF536* rs2053079 and creativity would not survive correction for multiple testing. Therefore, rather than hypothesis testing, the finding concerning the association between *ZNF536* rs2053079 and creativity should be more viewed as hypothesis generating, replication in independent samples of different ethnic populations is warranted.

Evidence from recent GWAS has revealed that most complex diseases and traits are highly polygenic and influenced by hundreds or even thousands of variants with small effects (Manolio et al., 2009; Gratten et al., 2014; Robinson et al., 2014). To account for the polygenic nature and to increase statistical power to detect weak associations of small effects, the polygenic score approach has recently been proposed and applied in genetic studies of complex diseases and traits (Purcell et al., 2009; Dudbridge and Wray, 2013). For example, the polygenic score approach has been used by the Psychiatric Genomics Consortium and the International Schizophrenia Consortium to investigate major depression and schizophrenia (Lee et al., 2013). And more recently, Power et al. (2015) demonstrated that the polygenic risk scores for schizophrenia and bipolar disorder would predict artistic society membership or creative profession. In this study, to test the accumulated effect of the 11 schizophrenia risk variants, the polygenic score approach was also employed to examine the association between the polygenic risk score of these risk variants and creativity. However, the results showed that the polygenic risk score was not associated with creativity, indicating limited contribution of the accumulated effect of these risk variants to creativity. But anyway, given the highly polygenic nature of schizophrenia and creativity, the polygenic score approach held the promise to fully elucidate the potential genetic link between schizophrenia and creativity, and should be encouraged in future studies.

Several limitations of this study should be considered. First, some important background information concerning the subjects that may bias the results was not included in this study. In this study, self-report history of psychiatric disorders was used as an inclusion criterion of the subjects; however, such exclusion criteria may be insufficient. Under such inclusion criteria (without structured interview), subjects with undiagnosed psychiatric disorders (e.g., undiagnosed mood disorders) known to affect creativity as well as subjects with relatives of psychiatric disorders patients were likely to be included in this study, which may confound the results. Moreover, other important variables (e.g., educational level and IQ level) that were likely to influence the relationship between schizophrenia risk variants and creativity were not included in this study. For future study, the inclusion of these important variables as well as the application of more strict inclusion criteria would help to clarify the findings of this study and lead to a more accurate interpretation of the results. Second, as mentioned above, due to the small sample size, this study may not provide adequate power to detect weak associations of very small effect sizes. According to power calculation of single genetic variant analysis, for common genetic variants (MAF 10%), our sample only has sufficient (80% power) to detect effect size (*R*^2^) greater than 0.0135 (with α = 0.05), thus the possibility of type I and type II error cannot be excluded. It is important for future studies to validate these results in larger samples. Third, only very limited numbers of schizophrenia risk variants were examined in this study, other crucial risk variants were not included. Since both schizophrenia and creativity are highly polygenic, the potential genetic link between schizophrenia and creativity may rely on complex networks and interactions of multiple risk variants. To better reveal the genetic contribution of schizophrenia risk variants to creativity, such complex networks and interactions should be considered and examined in future studies. Forth, only divergent thinking test was used in this study as a measure of creativity, which may not accurately reflect subjects’ real creativity. Although divergent thinking is critical to creativity, creativity itself is a multi-aspects concept and each aspect may represent a unique and complex trait. Besides divergent thinking, it is also necessary for future studies to investigate the associations between schizophrenia risk variants and other components of creativity (e.g., creative problem solving, creative personality, and creative achievement). Fifth, the potential interactions between schizophrenia risk variants and environment factors were not examined in this study. It has been well acknowledged that both schizophrenia and creativity are determined by the complex interplays of genes and environmental factors (Tsuang, 2000; Kandler et al., 2016). To fully elucidate the link between schizophrenia and creativity, it is reasonable for future studies to include environmental factors (e.g., education background and family environment) and focus on the potential effects of gene-environment interactions on the relationship between schizophrenia and creativity.

## CONCLUSION

In conclusion, this study provides limited evidence for the associations between these schizophrenia risk variants and creativity in healthy Han Chinese subjects. Future studies are warranted to better understand the potential genetic link between schizophrenia and creativity.

## Data Availability Statement

The raw data supporting the conclusions of this manuscript will be made available by the authors, without undue reservation, to any qualified researcher.

## Ethics Statement

This study was carried out in accordance with the recommendations of local ethics guidelines, Ethics Committee of Psychology Department of Capital Normal University with written informed consent from all subjects. All subjects gave written informed consent in accordance with the Declaration of Helsinki. The protocol was approved by the Ethics Committee of Psychology Department of Capital Normal University.

## Author Contributions

DW, QG, TG, and GeseDNA Research Team collected the data. DW, SZ, JZ, and JL were involved in the conception and design of the work. DW and SZ contributed in writing the main text of the manuscript.

## Conflict of Interest

TG and GeseDNA Research Team were employed by company Beijing Gese Technology Co., Ltd. The remaining authors declare that the research was conducted in the absence of any commercial or financial relationships that could be construed as a potential conflict of interest.
